# Anti-Obesity Activities of Chikusetsusaponin IVa and *Dolichos lablab* L. Seeds

**DOI:** 10.3390/nu10091221

**Published:** 2018-09-03

**Authors:** Jun Yin, Chang-Seob Seo, In Hyeok Hwang, Min Won Lee, Kwang Hoon Song

**Affiliations:** 1 Laboratory of Pharmacognosy and Natural Product-based Medicine, College of Pharmacy, Chung-Ang University, Seoul 156-756, Korea; yinjun89@naver.com (J.Y.); grampus92@naver.com (I.H.H.); 2 Herbal Medicine Research Division, 1672 Yuseong-daero, Yuseong-gu, Daejeon 34054, Korea; csseo0914@kiom.re.kr; 3 Korean Medicine Life Science, University of Science and Technology, 217 Gajeong-ro, Yuseong-gu, Daejeon 305-333, Korea

**Keywords:** *Dolichos lablab* L., chikusetsusaponin IVa, triterpenoid saponin, anti-obesity, 3T3-L1 cell differentiation

## Abstract

Obesity, a condition where excess body fat accumulates to the extent, causes a negative effect on health. Previously, we reported the extract of *Dolichos lablab* L. (DLL-Ex) inhibited high-fat diet (HFD)-induced increases in body weight and body fat mass and ameliorated increases in body weight. In the present work, we studyed the molecular mechanism for the inhibitory effect of DLL-Ex or Chikusetsusaponin IVa (CS-IVa), as isolated from *Dolichos lablab* L. (DLL) seeds extract, on adipocyte differentiation. We evaluated the effect of DLL-Ex, an anti-obesity agent, and CS-IVa, an active component of DLL-Ex, on 3T3-L1 cell differentiation via Oil red O assay and Q-PCR, along with their effects on CCAAT element binding protein alpha (C/EBPα), peroxisome proliferator-activated receptor gamma (PPARγ), fatty acid synthase (FAS), and fatty acid-binding protein 4 (FABP4) mRNA transcriptions. FAS and FABP4 protein expression levels after exposure to CS-IVa were also tested. The results showed that DLL-Ex and CS-IVa have potent inhibitory activity on adipocyte differentiation. Therefore, DLL and CS-IVa may be developed as a functional food material to treat obesity.

## 1. Introduction

*Dolichos lablab* L. (DLL), also known as hyacinth bean and part of the family Fabaceae, is widely grown in Africa and southern Asia, including in India and China [1]. DLL seeds are used for food and as a medicinal plant; it is reported that DLL is an effective agent against hypercholesterolemia, poison, gastrointestinal spasms, cholera, vomiting, diarrhea, leucorrhoea, and alcoholic intoxication [2,3,4]. Mature DLL seeds are also used for their antidiabetic, anti-inflammatory, analgesic, antioxidant, hypolipidemic, insecticidal, and antilithiatic activities, whereas the flower and leaf are used for their antimicrobial properties [5,6,7,8,9,10]. Previous studies have reported the isolation of chikusetsusaponin (CS)-IVa and lablabosides A, B, C, D, E, and F from DLL seeds [11]. Similarly, the purification of flavonoids, including cosmosiin, luteolin, luteolin-4′-*O*-*β*-d-glucopyranoside, luteolin-7-*O*-*β*-d-glucopyranoside, and rhoifolin from DLL flowers has been reported [12].

Obesity is a condition where excess body fat accumulates to the extent that it causes a negative effect on health. Specifically, obesity increases the risk of obstructive sleep apnea, type 2 diabetes, cardiovascular disease, certain types of cancer, depression, and osteoarthritis [13,14]. Indeed, the WHO reported in 2017 that obesity causes diabetes, cardiovascular disease, ischemic heart disease, colon cancer, and breast cancer. Therefore, it is important to treat obesity in order to prevent any subsequent diseases that would further damage the patient’s health [15]. In the majority of cases, obesity is caused by a combination of lack of physical activity, excessive food intake, and genetic susceptibility.

Another way to look at obesity is as an imbalance between energy intake and expenditure and between lipogenesis and lipolysis. One possibility of preventing obesity is through the prevention of lipogenesis. This would prevent the creation of adipocytes and fat accumulation, which in turn would minimize energy storage while increasing energy use. Adipocyte differentiation is a complex process that involves the coordinated expression of specific genes and proteins, such as peroxisome proliferator-activated receptor gamma (PPARγ) and CCAAT/enhancer binding protein alpha (C/EBPα) [16]. PPARγ and C/EBPα regulate the activation of lipogenic proteins, including fatty acid synthase (FAS) and fatty acid binding proteins (FABP) as well as lipid-specific genes necessary for the development of fat biosynthesis. The altered expression of PPARγ and C/EBPα, etc. could induce adipocyte differentiation, and the inhibition of adipocyte differentiation has been proved that was associated with prevention and treatment of obesity [17].

Many studies have explored the effects and potential mechanisms of anti-obesity and anti-diabetes therapies based on herbal extracts [18]. Our team recently reported that the extract of DLL (DLL-Ex) supplementation inhibited high-fat diet (HFD)-induced increases in body weight and body fat mass and ameliorated increases in body weight. The other studies revealed that CS-IVa, a main component of DLL-Ex, improved HFD-induced increased lipid levels in the serum and liver of mice [19,20]. However, the molecular mechanism for the inhibitory effect of DLL-Ex or CS-IVa on adipocyte differentiation has not yet been well clarified.

In the present work, for studying the molecular mechanism for the inhibitory effect of DLL-Ex or CS-Iva on adipocyte differentiation, we measured C/EBPα, PPARγ, FAS, and FABP4 mRNA transcriptions. FAS and FABP4 protein expression levels after exposure to CS-IVa were also tested. Finally, we also examined the content of CS-IVa in DLL. The results showed that the isolated triterpenoid saponin, CS-IVa, and DLL-Ex inhibited the differentiation and maturation of 3T3-L1 cells by inhibiting the expression of the adipogenic transcription factors PPARγ and C/EBPα. These results suggest that CS-IVa and DLL-Ex might be developed into a drug for the control nutraceutical treatment of obesity and related diseases.

## 2. Materials and Methods

### 2.1. Plant Material

DLL seeds were purchased from Kwangmyungdang (Ulsan, Korea) in March 2013 (origin; Myanmar) and were identified by Dr. Goya Choi, Herbal Medicine Research Division, Korea Institute of Oriental Medicine. A voucher specimen was deposited at the herbarium of the College of Pharmacy, Chung-Ang University (20160614DLL).

### 2.2. Extraction and Isolation

Dried DLL seeds (450 g) were extracted with 70% prethanol A (ethyl alcohol, which could be used for food) at room temperature to obtain DLL-Ex (56.81 g). DLL-Ex (55 g) was filtered through celite and isolated while using a Sephadex LH-20 column with a gradient elution system of H_2_O-MeOH (100:0 to 0:100, *v*/*v*) to obtain 21 fractions (Fr.1–Fr.21). Fr.10 was further separated using a Diasogel ODS-B column with a gradient elution system of H_2_O-MeOH (80:20 to 0:100, *v*/*v*) and then a Sephadex LH-20 column with a gradient of H_2_O-MeOH (60:40 to 0:100, *v*/*v*) to obtain CS-IVa (18 mg). CS-IVa was identified by using Nuclear Magnetic Resonance spectroscopy (NMR) (JEOL Nuclear Magnetic Resonance Spectrometers, 600 MHz, Peabody, MA, USA) and Mass spectroscopy (MS) (Thermo, Ultimate 3000 RSLC system and Thermo, Q Exactive, Waltham, MA, USA).

### 2.3. Ultra-Performance Liquid Chromatography (UPLC) Analysis

Quantitative determination of the bioactive compound, CS-IVa in DLL-Ex was performed while using a Waters Acquity UPLC^®^ H-Class system (Milford, MA, USA) equipped with an Acquity UPLC evaporative light scattering detector (ELSD). Data were acquired and then processed using Empower software (Waters Corporation, Milford, MA, USA). CS-IVa was separated on an Acquity UPLC C18 column (2.1 × 50 mm, particle size: 1.7 µm) at room temperature. The drift tube temperature and pressure of nitrogen in the ELSD were 60 °C and 40 psi, respectively. The mobile phase consisted of solvents A (0.1% formic acid in distilled water) and B (acetonitrile) that were previously filtered through Whatman^®^ membrane filters (0.22 µm, diameter. 47 mm). The gradient flow of the two mobile phases was as follows: 30% B at 0–0.5 min, 30–100% B at 0.5–5 min, and 100% B a 5–7 min. The injection volume was 0.4 μL and the flow rate was 0.5 mL/min.

### 2.4. 3T3-L1 Cell Culture and Differentiation

3T3-L1 preadipocytes (ATCC CL-173) were purchased from the American Type Culture Collection (ATCC, Rockville, MD, USA) and maintained in Dulbecco’s modified Eagle’s medium (DMEM), supplemented with 10% newborn calf serum (Gibco, Thermo Fisher Scientific, Waltham, MA, USA) and 1% antibiotic-antimycotic (Gibco). For adipocyte differentiation, the cells were seeded and cultured until confluent. Cells were then incubated for an additional 48 h with fresh culture media. The medium was switched to DMEM/F12 containing 10% fetal bovine serum (FBS; Gibco) and 1% antibiotic-antimycotic (Gibco) together with 1 µL differentiation cocktail (3T3-L1 differentiation kit, BioVision, Milpitas Blvd. Milpitas, CA, USA). After three days incubation with the differentiation medium, the medium was switched to DMEM/F12 containing 10% FBS and 1.5 µg/mL insulin for an additional four days. DLL-Ex and CS-IVa were added to the cell culture medium during the differentiation process. GW9662 (Sigma-Aldrich, St. Louis, MO, USA), an irreversible PPARγ antagonist, was used as an experimental control.

### 2.5. Cytotoxicity Assay

3T3-L1 cells were seeded in 48-well culture plates with DMEM supplemented with 10% newborn calf serum (Gibco) and 1% antibiotic-antimycotic (Gibco). The following day, medium containing DLL-Ex (at concentrations ranging from 10–500 μg/mL) and CS-IVa (at concentrations ranging from 1–50 μg/mL) were added. Twenty-four hours later, the cell proliferation rate was determined while using the CellTiter 96^®^ AQueous One Solution Cell Proliferation Assay (Promega, Fitchburg, WI, USA), according to the manufacturer’s protocol.

### 2.6. Oil Red O Staining Assay

Oil Red O staining assays were performed, as previously described [19]. Briefly, cells were washed twice with phosphate buffer solution (PBS), and fixed with 10% formalin for 1 h. Cells were then stained with Oil Red O solution and examined under a light microscope (Olympus). After observing the lipid droplets, 100% isopropanol was added to each well and the absorption intensity at 520 nm was measured with a spectrophotometer (Molecular Devices).

### 2.7. RNA Isolation and Quantitative Real-Time PCR

RNA was isolated from cells while using an RNeasy isolation kit (Qiagen), according to the manufacturer’s instructions. Reverse-transcription and Q-PCR using Taqman probes (ABI) were performed to detect relative mRNA expression, as described previously [19].

### 2.8. Protein Extraction and Western Blot Analysis

After differentiation, the cells were washed twice with ice-cold Dulbecco’s phosphate buffered saline (DPBS) and lysed in lysis buffer containing a protease inhibitor cocktail (Roche Applied Science). The cell lysates were kept in ice for 30 min and centrifuged at 14,000× *g* for 20 min at 4 °C. After centrifugation, the supernatants were removed and the protein concentrations in the supernatants were determined while using a BCA Protein Assay Kit (Thermo Scientific Pierce, Waltham, MA, USA). Western blotting was performed while using precast gels (Bio-Rad Laboratories, Hercules, CA, USA) and all the separated proteins were transferred onto polyvinylidene difluoride membranes (Bio-Rad Laboratories). The membranes were blocked with non-protein blocking reagent (Atto) for 30 min and then incubated with primary antibodies (Adipogenesis Marker Antibody Sampler Kit, Cell Signaling Technology) overnight at 4 °C. The membranes were washed with tris-buffered saline-tween (TBST) buffer and incubated with horseradish peroxidase-conjugated secondary antibodies (Cell Signaling Technology, Danvers, MA, USA) for 1 h at room temperature. The membranes were visualized while using an enhanced chemiluminescence (ECL) detection system (Thermo Scientific) and the bands were visualized using a chemiluminescence imaging system (Fusion Sl; Vilber Lourmet, Collégien, France).

### 2.9. Statistical Analysis

All data are presented as mean ± standard error of the mean (SEM). All data were tested by t-test analysis. All statistical analyses were performed using Statistical Product and Service Solutions (SPSS, SPSS Inc., Chicago, IL, USA) program (IBM, Armonk, NY, USA).

## 3. Results

**Figure 1:**
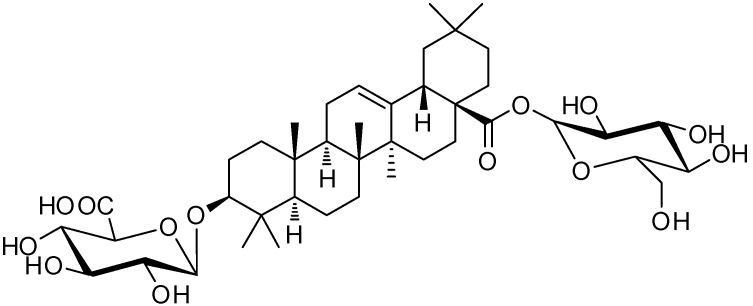
The chemical structure of Chikusetsusaponin IVa (CS-IVa).

CS-IVa was isolated from water soluble fraction of 70% prethanol A extract (Figure 1). The extract was separated via chromatographic isolation using a Sephadex LH-20 column and ODS-gel to isolate CS-IVa. CS-IVa showed a pink coloration when heated after spraying with 10% H_2_SO_4_ solution in thin layer chromatography (TLC). CS-IVa: White powder; HRFABMS *m*/*z* 793.4369 [M − H]^−^ (calculated for C_42_H_66_O_14_ 794.4447). ^1^H-NMR (600 MHz, Pyridine-*d*_5_) *δ* 6.26 (1H, d, *J* = 7.74 Hz, H_Glc_-1), 5.40 (1H, s, H-12), 4.95 (1H, brs, H_GlcUA_-1), 3.34 (1H, d, *J* = 11.4 Hz, H-3), 3.16 (1H, d, *J* = 11.76 Hz, H-18), 1.28 (3H, s, Me-23), 1.26 (3H, s, Me-27), 1.07 (3H, s, Me-25), 0.96 (3H, s, Me-24), 0.90 (3H, s, Me-26), 0.87 (3H, s, Me-29), 0.81 (3H, s, Me-30); ^13^C-NMR (125 MHz, Pyridine-*d*_5_) *δ* 176.82 (C-28), 176.78 (C_GlcUA_-6), 144.49 (C-13), 123.23 (C-12), 107.39 (C_GlcUA_-1), 96.09 (C_Glc_-1), 89.44 (C-3), 79.67 (C_Glc_-5), 79.18 (C_Glc_-3), 78.53 (C_GlcUA_-4), 77.66 (C_GlcUA_-3), 75.77 (C_GlcUA_-5), 74.43 (C_Glc_-2), 73.94 (C_GlcUA_-2), 71.07 (C_Glc_-4), 62.51 (C_Glc_-6), 56.12 (C-5), 48.34 (C-9), 47.34 (C-17), 46.55 (C-19), 42.47 (C-14), 42.07 (C-18), 40.25 (C-8), 39.85 (C-4), 39.00 (C-1), 37.29 (C-10), 34.36 (C-21), 33.54 (C-7), 33.53 (C-29), 32.87 (C-22), 31.15 (C-20), 28.61 (C-23), 26.50 (C-2), 28.58 (C-15), 26.88 (C-27), 24.12 (C-16), 24.01 (C-11), 23.74 (C-30), 18.84 (C-6), 17.81 (C-26), 17.35 (C-24), 15.91 (C-25). ^1^H-NMR spectrum of CS-IVa exhibited seven methyl signals [*δ* 1.28 (3H, s, Me-23), 1.26 (3H, s, Me-27), 1.07 (3H, s, Me-25), 0.96 (3H, s, Me-24), 0.90 (3H, s, Me-26), 0.87 (3H, s, Me-29), 0.81 (3H, s, Me-30)], and an olefin signal H-12 [5.40 (1H, s)], and the ^13^C-NMR spectrum of isolated compound showed one oleanolic acid group [*δ* 176.82 (C-28), 144.49 (C-13), 123.23 (C-12), 89.44 (C-3), 56.12 (C-5), 48.34 (C-9), 47.34 (C-17), 46.55 (C-19), 42.47 (C-14), 42.07 (C-18), 40.25 (C-8), 39.85 (C-4), 39.00 (C-1), 37.29 (C-10), 34.36 (C-21), 33.54 (C-7), 33.53 (C-29), 32.87 (C-22), 31.15 (C-20), 28.61 (C-23), 26.50 (C-2), 28.58 (C-15), 26.88 (C-27), 24.12 (C-16), 24.01 (C-11), 23.74 (C-30), 18.84 (C-6), 17.81 (C-26), 17.35 (C-24), and 15.91 (C-25)]. The ^1^H-NMR spectrum of CS-IVa showed two anomeric proton signals [*δ* 6.26 (1H, d, *J* = 7.74 Hz, H_Glc_-1), 4.95 (1H, brs, H_GlcUA_-1)] and the ^13^C-NMR spectrum of CS-IVa also showed the existence of a glucose and a glucuronic acid group [*δ* 176.78 (C_GlcUA_-6), 75.77 (C_GlcUA_-5), 78.53 (C_GlcUA_-4), 77.66 (C_GlcUA_-3), 73.94 (C_GlcUA_-2), 107.39 (C_GlcUA_-1) and 62.51 (C_Glc_-6), 79.67 (C_Glc_-5), 71.07 (C_Glc_-4), 79.18 (C_Glc_-3), and 74.43 (C_Glc_-2), 96.09 (C_Glc_-1)]. From these results, the structure of isolated compound was identified as Chikusetsusaponin IVa (CS-IVa), indicating the molecular formula as C_42_H_66_O_14,_ through a comparison with reference data [21].

**Figure 2:**
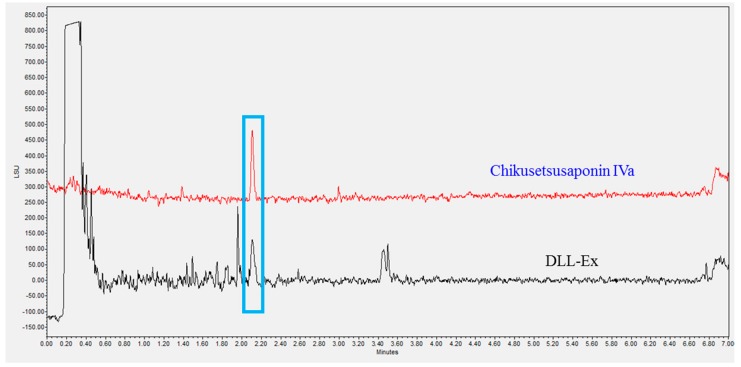
Ultra-performance liquid chromatography of CS-IVa and *Dolichos lablab* L. (DLL) extract.

The concentration of CS-IVa in DLL was analyzed via UPLC and the retention time of CS-IVa was found to be 2.08 min (Figure 2). The calibration curve for the quantitative analysis of CS-IVa in DLL was performed at a concentration range of 125–1000 μg/mL, and the concentration of CS-IVa in dried DLL was 614 μg/g (0.0614%) and in DLL-Ex was 5400 μg/g (0.54%).

**Figure 3:**
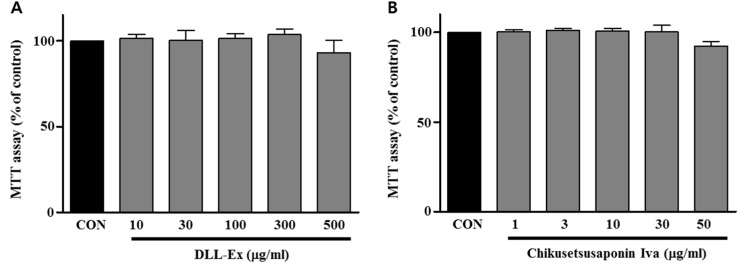
Effects of extract of DLL (DLL-Ex) and CS-IVa on the cell viability of 3T3-L1 preadipocytes. (**A**) MTT experiment of DLL-Ex; (**B**) MTT experiment of CS-IVa. The results were expressed as mean ± SEM of triplicate experiments.

Cytotoxicities of DLL-Ex and CS-IVa were determined using MTT assays. Figure 3 shows that DLL-Ex and CS-IVa had minimal cytotoxic effects up to 500 μg/mL and 50 μg/mL, respectively, in 3T3-L1 cells.

**Figure 4:**
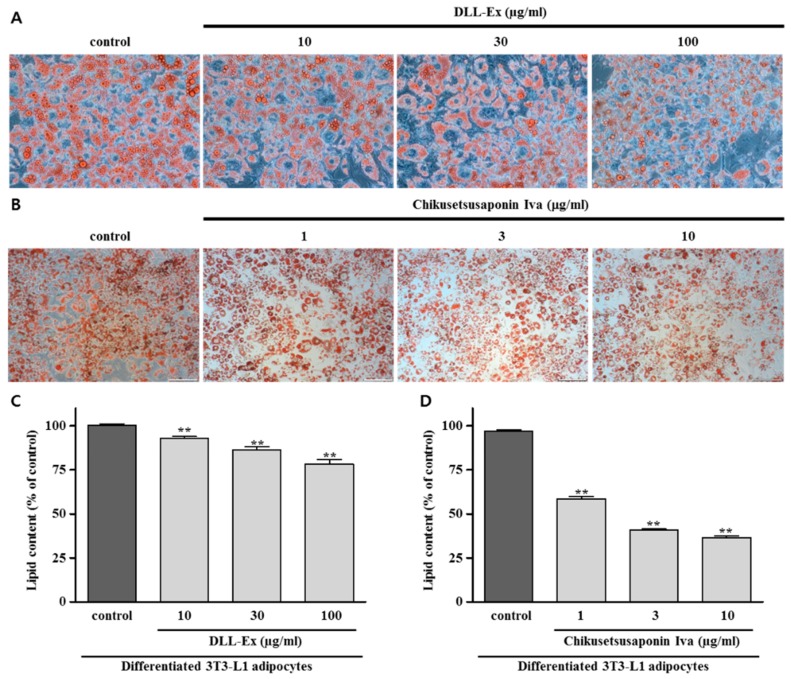
Effects of DLL-Ex and CS-IVa on lipid accumulation in 3T3-L1 adipocytes. (**A**,**C**) Inhibitory activity of DLL-Ex on lipid content; (**B**,**D**) Inhibitory activity of CS-IVa on lipid content. The results were expressed as mean ± SEM of triplicate experiments. ** *p* < 0.01.

To investigate the effect of DLL-Ex and CS-IVa on lipid formation, intracellular lipid accumulation was measured in differentiated adipocytes. Microscopic observations were used to identify the intracellular lipid droplets in differentiated adipocytes. Adipocyte differentiation of 3T3-L1 preadipocytes was examined using Oil red O staining (Figure 4). DLL-Ex and CS-IVa treatment significantly reduced intracellular lipid contents as compared to vehicle-treated differentiated adipocytes.

**Figure 5:**
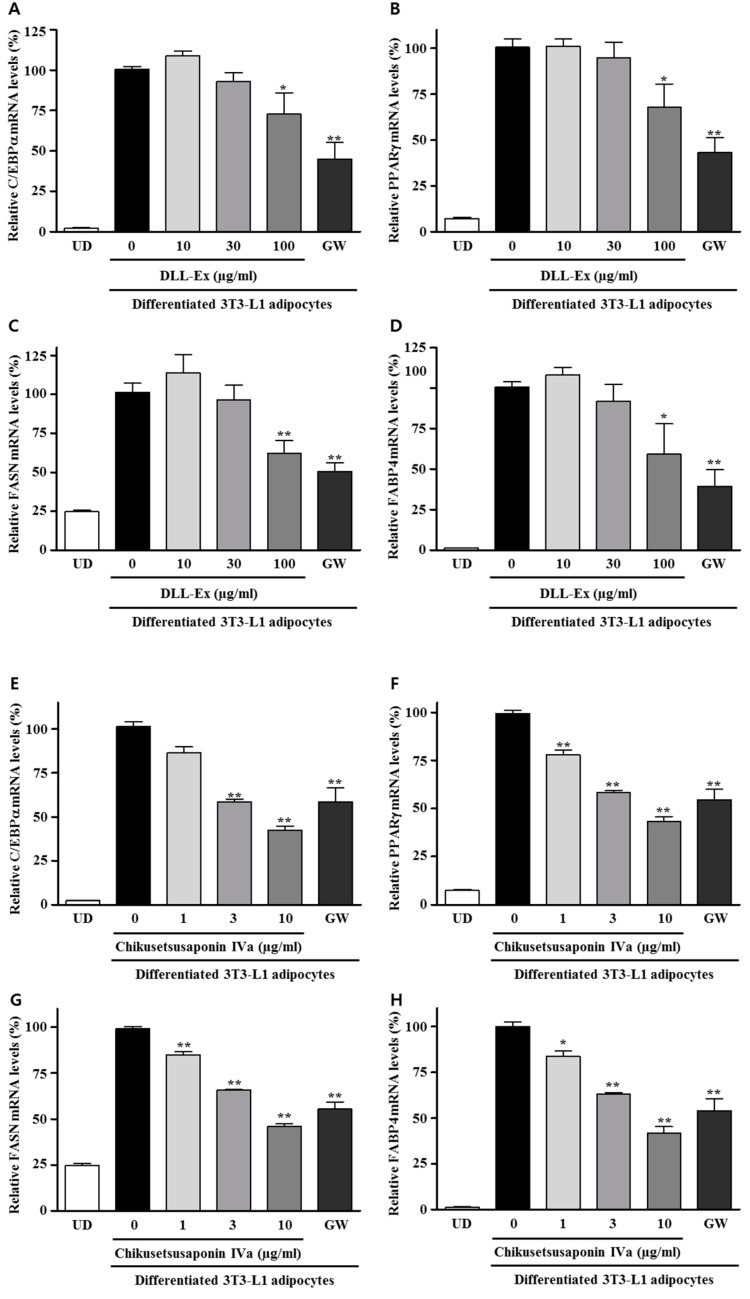
Effects of DLL-Ex and CS-IVa on mRNA expression of differentiation-related transcriptional factors and lipogenic genes in 3T3-L1 adipocytes. (**A**) Inhibitory activity of DLL-Ex on C/EBPα RNA levels; (**B**) Inhibitory activity of DLL-Ex on PPARγ RNA levels; (**C**) Inhibitory activity of DLL-Ex on FASN RNA levels; (**D**) Inhibitory activity of DLL-Ex on FABP4 RNA levels; (**E**) Inhibitory activity of DLL-Ex on C/EBPα RNA levels; (**F**) Inhibitory activity of DLL-Ex on PPARγ RNA levels; (**G**) Inhibitory activity of DLL-Ex on FASN RNA levels; (**H**) Inhibitory activity of DLL-Ex on FABP4 RNA levels. The results were expressed as mean ± SEM of triplicate experiments. ** *p* < 0.01, * *p* < 0.05. UD: Undifferentiated 3T3-L1 cells.

**Figure 6:**
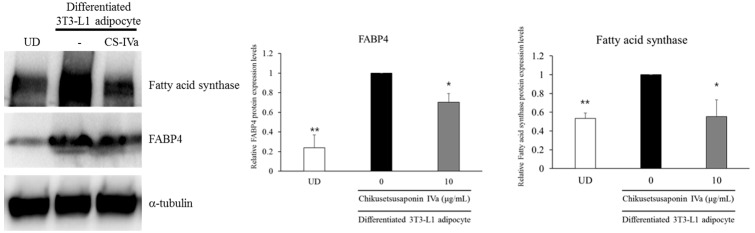
Effects of CS-IVa on protein expression of lipogenic genes in 3T3-L1 adipocytes. The results were expressed as mean ± SEM of triplicate experiments. ** *p* < 0.01, * *p* < 0.05. UD: Undifferentiated 3T3-L1 cells.

Further, we investigated whether DLL-Ex and CS-IVa affected C/EBPα, PPARγ, FASN, and FABP4 mRNA expression in differentiated adipocytes via Q-PCR (Figure 5). When compared with the vehicle-treated differentiated adipocytes, DLL-Ex and CS-IVa significantly inhibited the mRNA expressions of C/EBPα, PPARγ, FASN, and FABP4. Although low doses (10 and 30 μg/mL) of DLL-Ex showed no significant inhibitory effects on C/EBPα, PPARγ, FASN, and FABP4 mRNA expression, DLL-Ex significantly inhibited the mRNA levels of C/EBPα and PPARγ at a dose of 100 μg/mL. Moreover, CS-IVa inhibited the mRNA levels of C/EBPα, PPARγ, FASN, and FABP4 in dose-dependent manners. CS-IVa repressed the both mRNA and the protein expression of FAS and FABP4 (Figure 6).

## 4. Discussion

The 3T3-L1 cell line that is used in adipose tissue study could differentiate into mature adipocytes that are morphologically and biochemically similar to adipocytes [22,23]. The differentiated adipocytes were considered to be the key of obesity development [22,24,25]. For further research on anti-obesity of DLL-Ex or CS-Iva, in this study, we studied the molecular mechanism for the inhibitory effect of DLL-Ex or CS-Iva on adipocyte differentiation.

Lipid accumulation is one key feature in obesity. The beneficial effects of decreased lipid contents in the adipogenic differentiation of 3T3-L1 cells by DLL-Ex or CS-IVa suggest that DLL-Ex and CS-IVa inhibited the adipocyte differentiation effect.

C/EBPα and PPARγ are key activators of adipogenesis. They have been shown that they could directly enhance expression of adipocyte gene. C/EBPα is important for differentiation and maintenance of adipose and PPARγ is involved in adipogenesis, such as FABP4, LPL, and fatty acid transporter, and the maintenance of the adipocyte phenotype [26,27,28,29,30,31]. Our results showed that both DLL-Ex and CS-IVa showed inhibitory activity on C/EBPα and PPARγ expression. It is suggested that DLL-Ex and CS-IVa inhibited the differentiation and maturation of 3T3-L1 cells by inhibiting the expression of the adipogenic transcription factors PPARγ and C/EBPα.

FABP4, also called adipocyte protein 2 (aP2), is a carrier protein for fatty acid that is primarily expressed in adipocyte. FABP4 is similar to the FABPs family and are thought to enhance the transfer of fatty acids through binding to fatty acid [32,33]. Specially, FABP4 has been proved that FABP4 is expressed strongly induced by adipocyte differentiation [34], and FABP4 has been an adipocyte differentiation marker [35,36,37]. It is also reported that FABP4 and expression is controlled by C/EBP and PPARγ [38,39,40]. Since the expression of adipogenic transcription factors, C/EBPα and PPARγ, were down-regulated by CS-IVa, we further addressed the expression of their downstream genes, such as FAS and FABP4, which are important adipogenic proteins that are involved in fatty acid and triacylglycerol synthesis. These results suggest that CS-IVa represses preadipocyte differentiation and adipogenesis via inhibiting the expression of the adipogenic transcriptional factors and their downstream target genes.

Saponins are potential biological activity compounds that are insisted in various natural plants. Generally, saponins contains triterpenoid saponin and steroid saponin, that triterpenoids have C30 triterpene skeleton and steroid saponins have C27 steroidal skeleton. Some of saponins, especially triterpenoid saponins, are found as saponin glycosides that attach various sugar molecules with the triterpene skeleton, such as glucose, galactose. It is reported that triterpenoid saponins showed many biological activities, such as anti-microbial, hypolipidemic, immunomodulatory, anti-tumor, anti-diabetic, hypocholesterolemia, anti-coagulant, anti-carcinogenic, hepatoprotective, hypoglycemic, neuroprotective, anti-inflammatory, and anti-oxidant activity [41,42,43]. Some of them and saponin-enriched extract also showed excellent anti-obesity activity [44,45,46,47,48,49,50,51,52,53,54]. CS-IVa also showed good anti-hyperglycemic, hypolipidemic, anti-inflammatory, cardioprotective, and neuroprotective activities [20,55,56,57]. At present, CS-IVa and saponin-enriched DLL-Ex proved that good adipocyte differentiation agents, through their inhibitory activities on PPARγ, C/EBPα, FAS, FABP4, and decreasing the activity of lipid accumulation. Thus, CS-IVa may a key bioactive compound in DLL-Ex induces adipocyte differentiation.

Our recent animal study revealed that DLL-Ex not only repressed hepatic steatosis but also repressed weight gain by HFD [19]. Present work showed that DLL-Ex and its active constituent CS-IVa inhibit adipocyte differentiation and decrease lipid accumulation. These results suggest that the anti-obesity effect of DLL-Ex likely resulted from the inhibition of adipogenesis via CS-IVa.

## 5. Conclusions

CS-IVa, which is a bioactive component of DLL-Ex (0.54% in DLL-Ex), decreased the expression of genes that are important to the adipogenesis process in 3T3-L1 adipocytes, suggesting that it is the active ingredient that suppresses the adipocyte size, number, and intracellular fat accumulation found after treatment with DLL-Ex. The biological availability concentration of CS-IVa is 16.29 mg/mL in raw seeds, or 1.85 mg/mL in DLL-Ex. These results suggest that CS-IVa and DLL-Ex might be developed into a drug for the treatment of obesity and related diseases.
